# Supplementary figures and images for: Identification and Structural Aspects of G-Quadruplex-Forming Sequences from the Influenza A Virus Genome
Source: Int J Mol Sci. 2021 Jun 2;22(11):6031. doi: 10.3390/ijms22116031 (PMC8199785; doi:10.3390/ijms22116031)

**a**

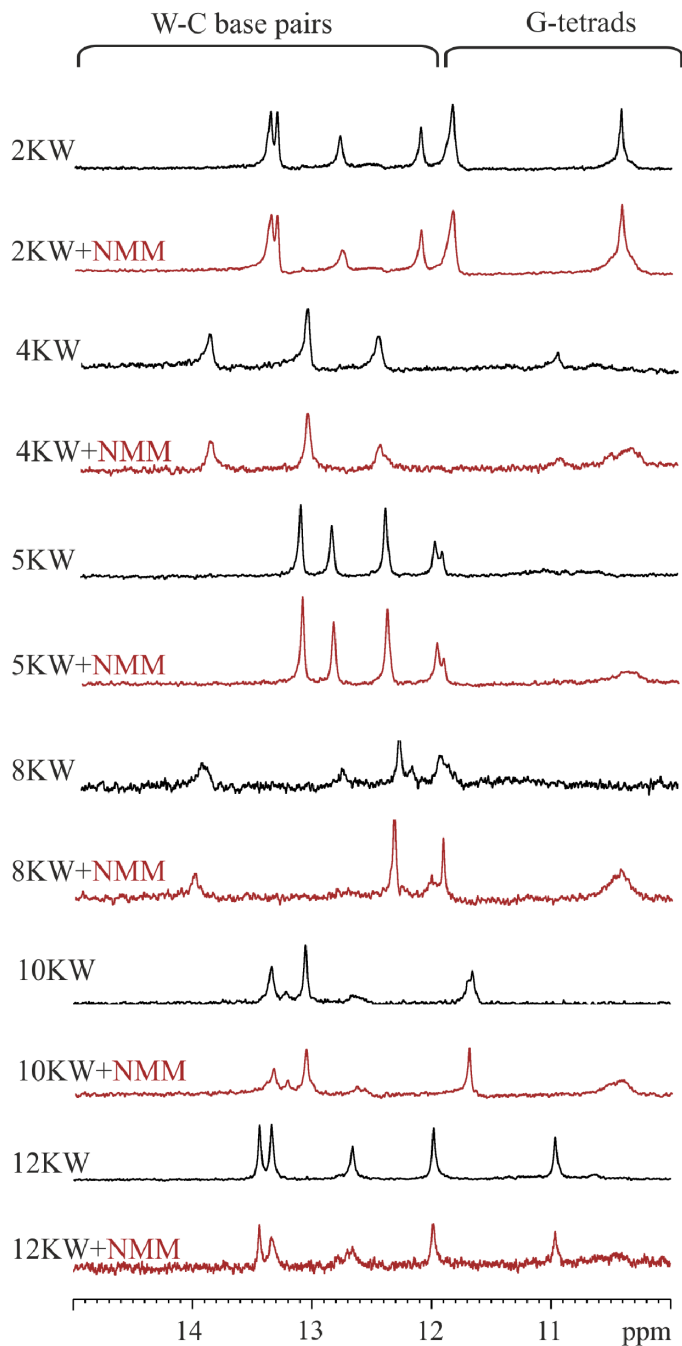**b**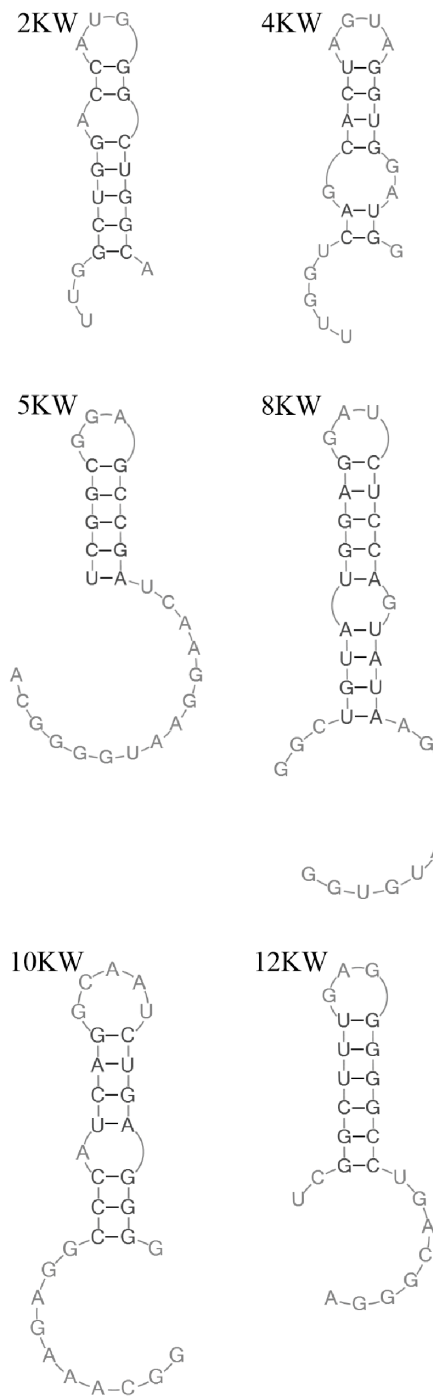

Supplement: Supplementary file 1 [file ijms-22-06031-s001.zip › ijms-1213014-supplementary.pdf]
